# Cost-effective isolation of *Viburnum opulus*-derived nanovesicles and evaluation of their cytotoxic, anticancer, and antioxidant properties on human glioblastoma cell line U87MG

**DOI:** 10.1007/s12032-025-02669-6

**Published:** 2025-03-17

**Authors:** Nazli Irmak Giritlioglu, Fatma Sayan Poyraz, Banu Mansuroglu, Semiha Erisen

**Affiliations:** 1 Department of Molecular Biology and Genetics, Graduate School of Science and Engineering, Yildiz Technical University, Istanbul, Turkey; 2 Department of Molecular Biology and Genetics, Faculty of Arts and Science, Yildiz Technical University, Istanbul, Turkey

**Keywords:** Exosome, Glioblastoma, Nanovesicle, Plant-derived nanovesicle, *Viburnum opulus*

## Abstract

Glioblastoma is the most common and highly invasive glial tumor, significantly reducing patient survival. Current therapeutic approaches have limited success rates. Plant-derived nanovesicles are a rapidly developing area, recognized for their exceptional biofunctional properties, and are emerging as a promising approach in cancer treatment. The present study focuses on the isolation of nanovesicles from *Viburnum opulus* fruits using a cost-effective method that includes a polymer-based exosome precipitation buffer and size exclusion chromatography, followed by their characterization. Morphological analysis via Field Emission Scanning Electron Microscopy and Transmission Electron Microscopy revealed nanovesicles ranging from oval to elliptical shapes, with average diameters of 54.23 nm and 41.21 nm, respectively. Dynamic light scattering analysis determined the average size of 45.36 nm indicating the presence of nanovesicles, and the zeta potential was − 2.87 mV. Biochemical characterization showed total protein and phenolic concentrations of 1534 ± 97.78 µg/ml and 4.270 ± 0.66 mg gallic acid equivalents/L, respectively, with total antioxidant status values of 3.83 ± 0.37 mmol Trolox equivalents/L. Based on IC50 values, these nanovesicles were 7.5 times more toxic to U87MG human glioblastoma cells compared to healthy human dermal fibroblasts. Analyses including clonogenic cell survival, wound healing, apoptosis, total antioxidant status, and total oxidant status were continued on only U87MG cells, as human dermal fibroblasts showed a low response to nanovesicle treatment. Qualitative and quantitative assessments demonstrated that *Viburnum opulus*-derived nanovesicles effectively inhibited cancer cell proliferation and migration. Due to their non-toxic, anticancer, and antioxidant properties, these nanovesicles hold significant potential in glioblastoma management.

**Graphical abstract:**

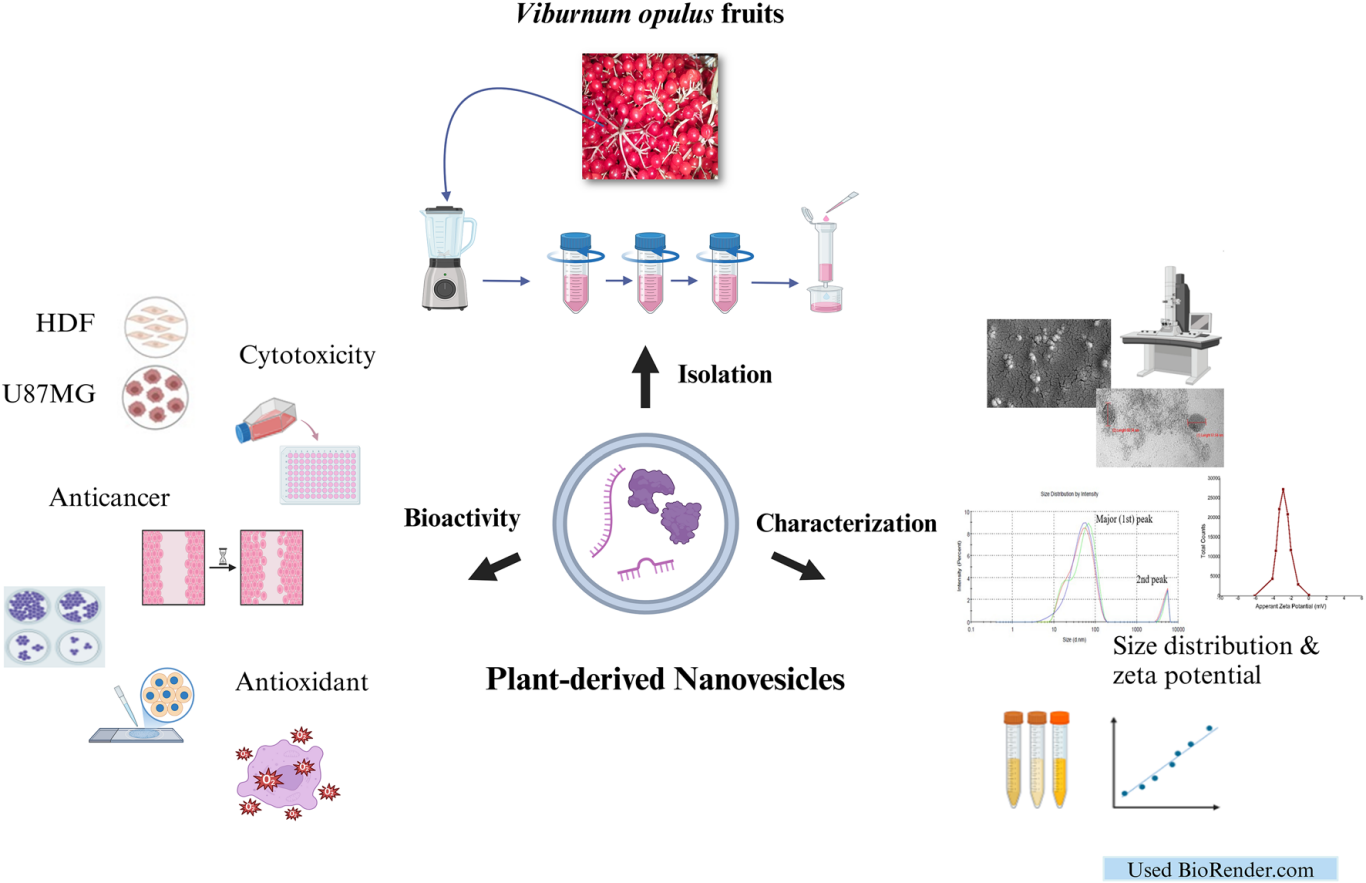

## Introduction

Glioblastoma (GB) is one of the treatment-resistant primary malignant brain tumors and accounts for about 54% of gliomas. The median survival time is 15 months, and the treatment process usually includes radiotherapy and chemotherapy sessions after tumor removal [1]. The primary challenge in GB treatment is the effective delivery of therapeutics to increase their efficacy and bioavailability [2]. New methods for drug delivery and release are essential. While innovative biotechnological solutions, such as non-pathogenic viruses, have been proposed to treat GB, challenges like immune system response and dose limitation also arise. Viral drug delivery and optimal dosing must be tested meticulously, and the appropriate virus must be tailored for each patient [3]. Plant-derived nanovesicles (PDNVs) have recently emerged as promising agents in cancer treatment.

PDNVs are the type of extracellular vesicles isolated from fruits or other various plant parts. They are nano-sized (30–150 nm) and considered in the exosome-like category. In the literature, various terms are utilized to describe PDNVs, including “plant-derived edible nanoparticles” [4], “plant-derived exosome-like nanoparticles” [5], “plant-derived exosomes” [6], and “plant-derived extracellular nanovesicles” [7]. PDNVs have important roles in the development of plants and protection from pathogens, cell-to-cell communication, and homeostasis [8, 9]. Some researchers describe them as rising stars, especially in medicine [10]. PDNVs can be isolated from plant sources in many ways. The first step is always to prepare juice or extract. The isolation method using low-speed centrifugation, ultracentrifugation (UC), and density gradient is accepted as the gold standard [5]. Different isolation techniques such as ultrafiltration, commercial kits, polyethylene glycol precipitation, immunoaffinity capture, electrophoresis-dialysis, size exclusion chromatography (SEC), and combinations of these methods are also possible. Polymer precipitation is a simple chemical process based on the separation of the biomolecule with reduced solubility from the mixture. SEC, on the other hand, provides rapid and effective isolation of biomolecules with desired properties by applying the sample to a porous filling material.

PDNVs have many advantages over synthetic nanoparticles. They are naturally non-toxic, safe, stable, and environmentally friendly. Their cellular uptake rate is higher compared to synthetic liposomes [11, 12]. It is also possible to encapsulate desired cargos in PDNVs and target them to tissues and organs. Since they are in a double-layered membrane structure, their contents can be protected from temperature and pH. Studies in this field have been increasing in recent years especially due to their biocompatibility and large-scale production features [13].

*Viburnum opulus* is a plant belonging to the *Adoxaceae* family, genus *Viburnum L.* that can grow in Europe, Russia, North Asia, and North Africa. This plant can be used for medicinal, food, and decorative purposes. *V. opulus* is also known as snowball tree, guelder rose, rose elder, cherry wood, water elder, and gilaburu. The fruit of the plant has a vinegar-like odor and a sour taste. The most popular use of the fruit among the public as a folk remedy for treating ureteral stones has attracted the attention of researchers. *V. opulus* has also antiobesity, antidiabetic, antimicrobial, anti-inflammatory, antioxidant, and anticancer properties [14]. Various volatile organic compounds have been identified in *V. opulus* fruits, including terpenoids, alcohols, ketones, phenols, esters, branched fatty acids, aldehydes, and other acids. Phenolic compounds have essential functions in scavenging free radicals (antioxidant activity), signaling mechanisms, xenobiotic metabolism, and gene and protein expression. Antiproliferative and anti-angiogenic (anticancer) effects of *V. opulus* have been evaluated in many different cell lines such as human cervical carcinoma (HeLa), lung cancer (A549 and Caco-2) [15], and colorectal adenocarcinoma (HT-29). The findings revealed that *V. opulus* fruit pomace effectively suppressed the proliferation of cancer cells while sparing normal epithelial cells [16]. The inhibitory effect is credited to the diverse bioactive phytochemicals present in the fruits, including catechins, anthocyanins, procyanidins, and quercetin derivatives. *Trans*-*p-*coumaric acid, a hydroxy derivative of cinnamic acid is one of the most important active compounds of *V. opulus* and creates anticarcinogenic effects in colorectal cancer cells, DLD-1 [17]. As is known, a decrease in proapoptotic proteins is observed in uncontrolled proliferating cancer cells. In one of the interesting studies, researchers proved that *V. opulus* extract leads to an increase in gene expression associated with activating the proapoptotic pathway in prostate cancer cells, LNCaP [17]. The juice extract was also found to inhibit the growth of tumor cells in a study on the Ehrlich ascites tumor model in mice [18].

In this study, we aimed to isolate *V. opulus*-derived nanovesicles (VDNVs) by polymer precipitation and SEC, to characterize them physically and biochemically, and to evaluate their cytotoxic, anticancer, and antioxidant effects on GB cells. NVs have not been isolated from *Viburnum* species before and the cost-effective method used in the study is easy to apply in every laboratory.

## Materials and methods

### Plant material

*V. opulus* fruits used in VDNV isolation were purchased from a registered farming company (Gilaburu Meyvesi, Hisarcık/Kayseri, Turkey) during the autumn harvest. Orange-colored fruits were discarded, and only red ones were used in the experiments. Fruits were stored at − 20 °C until use.

### Isolation of VDNVs

VDNV isolation steps before SEC were similar to Unsal et al.’s method [18]. Fruits were washed with distilled water, juiced with a 1000-W juicer (AR191 Meyvix, Arzum, Turkey), and centrifuged at varying speeds (1000 × g for 15 min, 3000 × g for 25 min, and 5000 × g for 90 min). The supernatant was collected each time and filtered using sterile 0.45- and 0.22-µm filters (Macherey–Nagel, Germany). Exosome precipitation buffer (Exo-spin, Cell Guidance Systems, USA) was added to the supernatant at a supernatant:buffer ratio of 2:1. The mixture was stored at 4 °C overnight and centrifuged at 5000 × g for two hours. The supernatant was removed, fresh 1X phosphate-buffered saline (PBS) (pH 7.4) was added, and SEC was applied to isolate VDNVs with 30–150 nm diameters. The columns were washed with PBS, and the collected fractions were filtered and stored at − 20 °C for subsequent experiments.

### Field emission scanning electron microscopy (FESEM) and transmission electron microscopy (TEM) imaging

FESEM analysis was conducted at the Central Research Laboratory Application and Research Center, Eskisehir Osmangazi University (ARUM), Turkey. The sample was coated using EM ACE600 sputter coater (Leica Microsystems, Germany). Regulus 8230 (Hitachi, Japan) was used for morphology and size analysis. Imaging was performed at voltages between 3 and 20 kV and magnifications ranging from 10,000 to 500,000 ×. TEM analysis was also carried out at ARUM. 1220 JEM (JEOL, Japan) was used for detailed surface topography, morphology, and VDNV size analysis. Imaging was performed at 100-kV voltage and magnifications ranging from 80,000 to 200,000 × were applied. For FESEM and TEM analyses, experiments were conducted with at least three technical replicates, utilizing a mix of three distinct VDNV samples.

### Size and zeta potential analysis

Dynamic Light Scattering (DLS) analyzer (Zetasizer Nano ZS, Malvern, UK) was used to measure the Z-average, polydispersity index (PDI), size distribution by intensity, and zeta potential of VDNVs. PBS was used as a dispersant. The experiments were conducted at 25 °C, using a mix of three different VDNV samples and performing three technical repeats. The viscosity was 6.4475 cP and the refractive index was 1.347 for the solution. Analyses were carried out at Yildiz Technical University Central Research Laboratory, Turkey.

### Total protein concentration

A highly sensitive (0.5 μg/ml) micro Bicinchoninic acid (microBCA) protein assay kit (SK3061, Bio Basic, Canada) was used to determine the total protein concentration of VDNVs. This colorimetric assay depends on the reduction of Cu^2+^ to Cu^+^ by protein in an alkaline medium. A standard curve was created based on the absorbance at 562 nm of diluted bovine serum albumin concentrations (0 to 1000 μg/ml) and the total protein concentration of VDNVs was determined using this curve. Total protein concentration and VDNV concentration were considered directly proportional. The final concentration was adjusted to 1000 μg/ml to investigate the cellular effects of VDNVs.

### Total phenolic content (TPC)

VDNVs were mixed with methanol (Dolar Kimya, Turkey), then the mixture was vortexed and incubated at room temperature for 10 min and centrifuged at 5000 × g for 15 min. The supernatant fraction was mixed with the tenfold diluted Folin–Ciocalteu reagent (Merck, USA) and vortexed. pH was adjusted to 10 with 7.5% sodium carbonate (Kimyalab, Turkey) solution and the sample was incubated at room temperature for 30 min. The standard curve graph was obtained using gallic acid (Merck, USA) standards. Color change was measured at 765 nm in a spectrophotometer and the TPC was calculated.

### Total antioxidant status (TAS)

A commercial kit (Rel Assay Diagnostics, Turkey) based on 2,2'-azino-bis(3-ethylbenzothiazoline-6-sulfonic acid) (ABTS) reduction of antioxidant molecules was used to determine the TAS of the VDNVs. Spectrophotometric measurements were performed at 660 nm. The results were calculated as mmol Trolox equivalent (eqv.)/L.

### Cell culture

Cell culture studies were carried out similarly to the standard protocol [19, 20]. U87MG human GB (cancer cell line, ATCC code: HTB-14) and healthy human dermal fibroblast (HDF, ATCC code: PCS-201–012) were used in the in vitro studies of VDNVs. DMEM supplemented with 10% fetal bovine serum (FBS), streptomycin (100 µg/mL), penicillin (100 U/mL), and L-glutamine (0.2 mM) (Gibco, USA) was used in cell cultures. A humidified incubator at 37 °C with 5% CO_2_ provided physical conditions for cell growth. Passaging was ensured when cells covered 80–90% of the flask bottom by daily microscopic examination.

### In vitro cytotoxicity

The 3-[4,5-dimethylthiazol-2-yl]-2,5 diphenyl tetrazolium bromide (MTT) assay [21], considered the gold standard in cytotoxicity studies, was used to evaluate the impact of VDNVs on cell viability. U87MG and HDF cells were grown in the 96-well plates at a seeding density of 1 × 10^4^ per well. After incubating the cells overnight, they were treated with VDNV at different concentrations (0–1000 µg/mL) and incubated for 24 and 48 h. The culture medium was removed and 50 µl of MTT (Bio Basic, Canada) solution was added to each well and incubation was performed for 3 h to observe formazan crystals. Then, 100 µl of dimethyl sulfoxide (Merck, USA) was added to dissolve the formazan products and the absorbances measured at 570 nm in a microplate reader were recorded. The experiment was repeated three times. The following formula was used to calculate the percentage of cell viability:

$$\text{Cell viability \%}  =\left(\frac{\text{Optical density value of treated cells}}{\text{Optical density value of control cells }}\right)\text{x 100}.$$

### Clonogenic cell survival assay

Optimized colony formation [22] experiments were repeated three times for VDNV and the control group. U87MG cells were seeded in a 6-well microplate at 1 × 10^4^ cells/well. IC50 doses of VDNVs were added and the cells were incubated for 24 and 48 h. After incubation periods, cells were collected using trypsin and centrifuged. After seeding a maximum of 100 cells per well in 6-well plates, the cells were allowed to grow for 7–8 divisions. The medium was changed every three days. After a 10-day incubation period, the cells were rinsed with 1X PBS and then fixed with ice-cold methanol for 10 min. They were stained with 0.2% crystal violet for 15 min, making the formed colonies visible. The culture dishes were rinsed 2–3 times with distilled water to remove any dye remnants. The stained colonies were counted, and the survival percentage was graphically represented.

### Wound healing assay

The wound healing assay, also known as the in vitro scratch test, was performed similarly to the protocol in the literature [23]. U87MG cells were seeded at a density of 1 × 10^5^ cells/well in 24-well microplates and grown to 90% confluence. Then, a physical wound was created along the center of the cell monolayer using a sterile pipette tip. Following this procedure, IC50 doses of VDNVs were applied to the cells. The experiment was performed in triplicate. Microscopic monitoring was performed on cells allowed to migrate in FBS-free medium for 24 and 48 h, and wound closure gaps were calculated.

### DAPI staining for apoptosis analysis

An optimized protocol [24] was used for the apoptosis analysis of GB cells. U87MG cells were seeded into sterile 6-well plates as 1 × 10^5^ cells/well. 2 mL of medium was added, and the cells were incubated for 24 h in a 37 °C incubator containing 5% CO_2_. After the cells were treated with IC50 doses of VDNVs, incubation was performed for 24 and 48 h. VDNVs were not applied to the control group.

The DAPI reagent (Sigma-Aldrich, USA) at a concentration of 50 µg/ml, prepared in 1X PBS, was applied to both the control and VDNV groups at the end of the incubation periods. The entire procedure was conducted in darkness to preserve the reagent’s integrity. DAPI-treated cells were incubated for 10 min in a 37 °C humidified incubator with 5% CO2 to ensure proper binding to their specific targets. Following incubation, the DAPI was removed from the cells with stained nuclei, and any excess dye was washed away with 1X PBS. The wells were then observed under a fluorescence microscope (AxioVert A1, Carl-Zeiss, Germany) using 20X magnification and appropriate filters. The mean fluorescence intensity of apoptotic cells in the control group was calculated. In the VDNV treatment group, nuclei exhibiting fluorescence intensity above this threshold were classified as apoptotic, and these cells were subsequently counted and subjected to quantitative analysis. Apoptosis rates were calculated for both the control and VDNV groups and a graph was created by counting the number of apoptotic nuclei.

### In vitro TAS and total oxidant status (TOS)

T25 flasks were used for seeding U87MG cells and a concentration of 1 × 10^6^ cells per flask was applied. After the cells reached confluence, VDNVs were added and incubated for 24 and 48 h. Then, the cells were dislodged with trypsin, and the lysates were centrifuged at 1000 rpm for 5 min. The supernatant was removed, and 1 mL of cold PBS was added to the cell lysates for analysis.

A commercial ABTS-based colorimetric test kit (E-BC-K801-M, Elabscience, USA) was used to calculate the TAS values ​​of VDNVs in cell lysates. Serial dilution of the standard solution was prepared with 60% ethanol (Merck, USA) between 0–2 mmol/L and a standard curve was created. The absorbance change at 660 nm was recorded. Likewise, a colorimetric commercial kit (E-BC-K802-M, Elabscience, USA) was preferred for the TOS experiments. The maximum absorption wavelength is approximately 590 nm, and the color depth and content are proportional. Then, the hydrogen peroxide (H_2_O_2_) standard dilutions in volumes of 0–100 μmol/L were prepared with double distilled water and a calibration curve was generated. The formulas based on the optical density value of the sample were used to calculate the sample concentration in both procedures.

### Statistical analysis

GraphPad Prism (GraphPad Software LLC, Boston, MA, USA) was used for statistical analyses of biochemical characterization and cell culture studies that were performed with at least three biological replicates (n) and data were presented as mean ± standard deviation (SD). Simple linear regression was used in the biochemical characterization at a 95% confidence interval band. Unpaired *t* tests were used to evaluate comparisons between groups and *p* < 0.05 was considered statistically significant in cell studies.

## Results

### Isolation and characterization of VDNVs

**Fig. 1:**
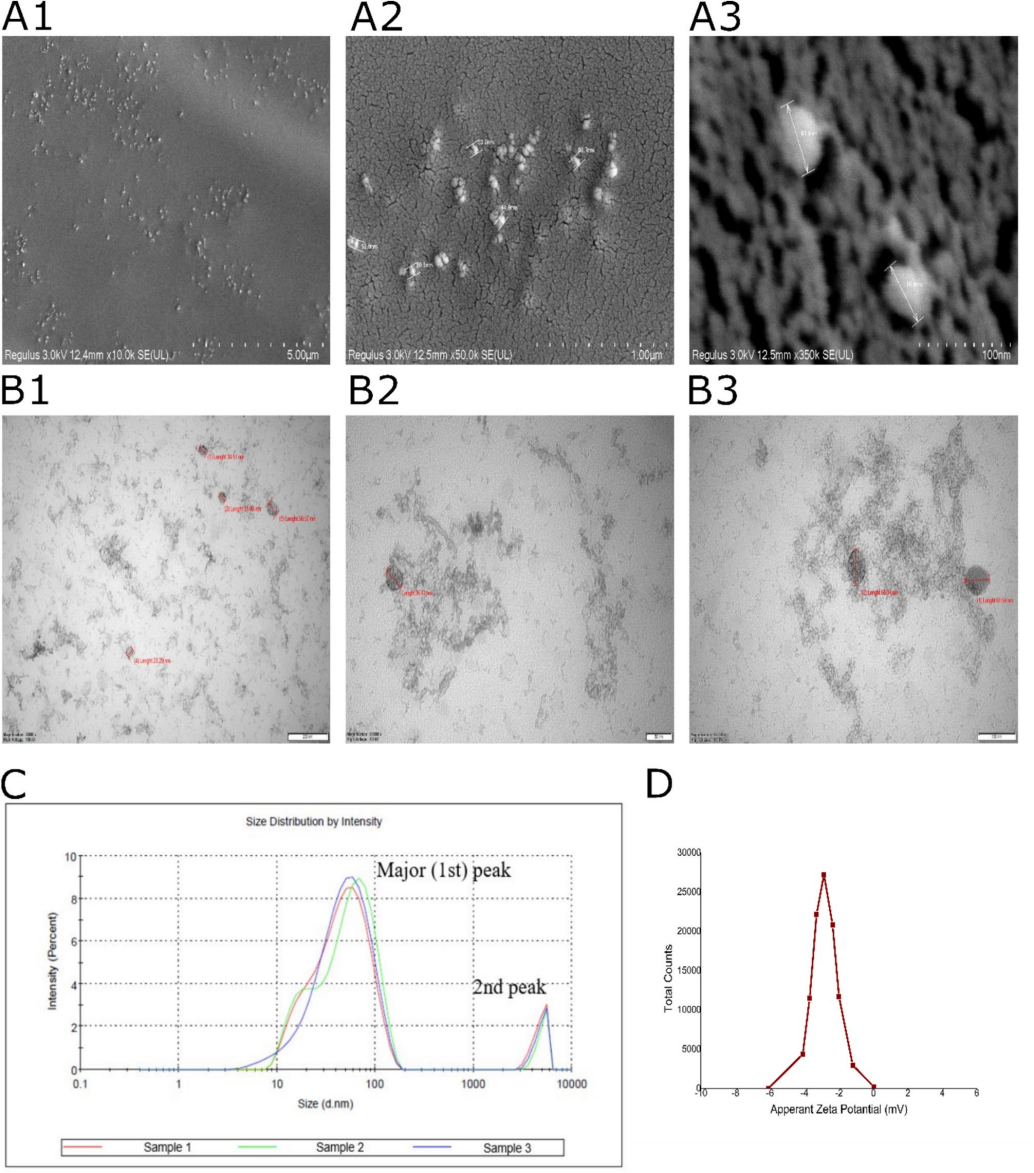
Physical characterization of VDNVs. In the top row are FESEM images labeled **A1** (scale bar: 5 µm), **A2** (scale bar: 1 µm), and **A3** (scale bar: 100 nm), while in the bottom row are TEM images labeled **B1** (scale bar: 200 nm), **B2** (scale bar: 50 nm), and **B3** (scale bar: 100 nm). The particle size distribution analysis was performed using DLS, as illustrated in graph (**C**) below, alongside the zeta potential analysis (**D**) of VDNVs. VDNVs (first peak) and aggregations (second peak) can be seen in the intensity/size graph. All experiments include three technical replicates using a mixture of three different VDNV samples obtained by SEC

A polymer-based commercial exosome precipitation buffer was used during VDNV isolation. After centrifuging, the resulting pellet was light pink, jam-like, and had a vinegar-like odor due to the nature of the *V. opulus* fruits. FESEM (Fig. 1A) and TEM (Fig. 1B) were used for the morphological analysis of VDNVs. According to the analysis, the existing VDNVs varying from oval to ellipse shapes were proven. The diameters of VDNVs were between 37.5 nm and 63.9 nm in FE-SEM, while they ranged from 13 to 66.34 nm in TEM. The average diameter for well-distinguishable VDNVs was 54.23 nm and 41.21 nm for FESEM and TEM, respectively.

DLS analysis was performed to determine the VDNV diameter after SEC. The results showed that the Z-average was 45.36 d.nm and PDI was 0.361. Two different peaks were observed in the intensity/size graph. Exosomes with a diameter of 54.09 ± 30.34 nm at 93.2% were seen in the first (major) peak, while the other structures with a diameter of 4899 ± 671.7 nm at 6.8% were detected in the second peak (Fig. 1C). The zeta potential (Fig. 1D) of VDNVs was found to be − 2.87 mV and the conductivity was 11.9 mS/cm.

As previously described, microBCA, Folin–Ciocalteu, and TAS assays were applied for the biochemical characterization of VDNVs. Total protein and phenolic concentrations of VDNVs were found as 1534 ± 97.78 µg/ml and 4.270 ± 0.66 mg gallic acid eqv./L, respectively. The TAS result was 3.83 ± 0.37 mmol Trolox eqv./L.

### Cell culture studies

#### In vitro cytotoxicity

**Fig. 2:**
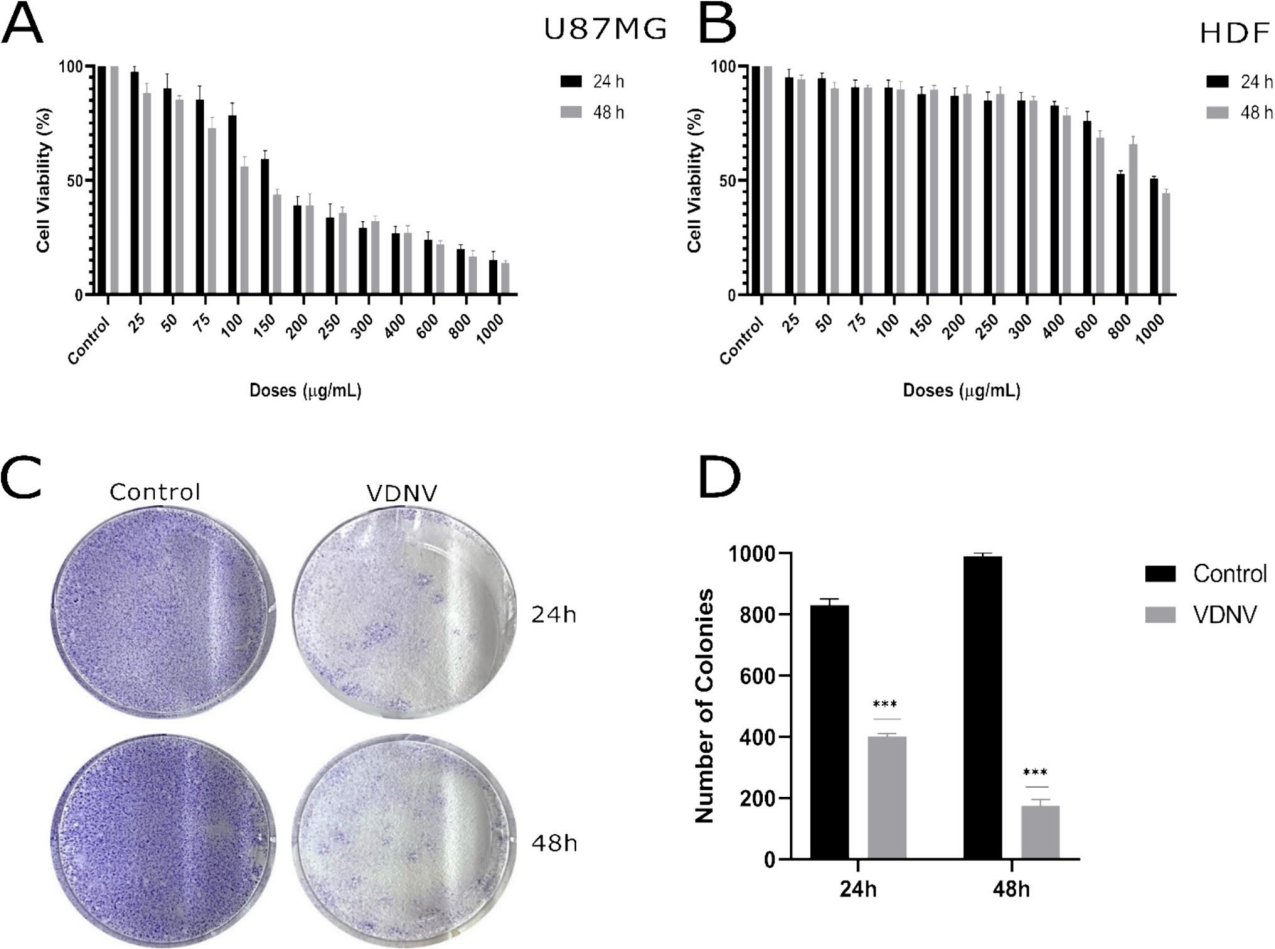
Cytotoxic activity of VDNVs on U87MG (**A**) and HDF cells (**B**) was determined by calculating the IC50 values. Representative images of colony formation (**C**) and quantitative analysis of colony numbers (**D**) in control and VDNV-treated U87MG cells at 24 and 48 h. All experiments were conducted with three independent biological replicates (*n* = 3). ***p < 0.001

The cytotoxic effect of VDNVs on U87MG and HDF cell lines at concentrations ranging from 0 to 1000 µg/mL was determined by MTT assay. The IC50 value of VDNVs in U87MG cells for 24 h was defined as 173.20 µg/mL, while that for 48 h was 125.86 µg/mL (Fig. 2A). In contrast to the low IC50 doses determined in U87MG cells, VDNVs inhibited only 48% of HDF cells at the highest dose of 1000 µg/mL after 24 h. The IC50 value for 48 h was determined as 948.12 µg/mL (Fig. 2B). Since there was no lethal cytotoxic effect for HDF cells in the given IC50 dose range, all other cell experiments were performed with U87MG cells only.

#### Clonogenic cell survival

The clonogenic cell survival assay, which was used to evaluate the effects of VDNVs on proliferation in U87MG cells, has been previously described. A qualitative observation in Fig. 2C shows that VDNVs applied to U87MG cells at IC50 doses suppressed colony formation. Colony formation was observed as 48.19% at 24 h for the VDNV group, while it was 17.67% at 48 h (Fig. 2D).

#### Wound healing assay

**Fig. 3:**
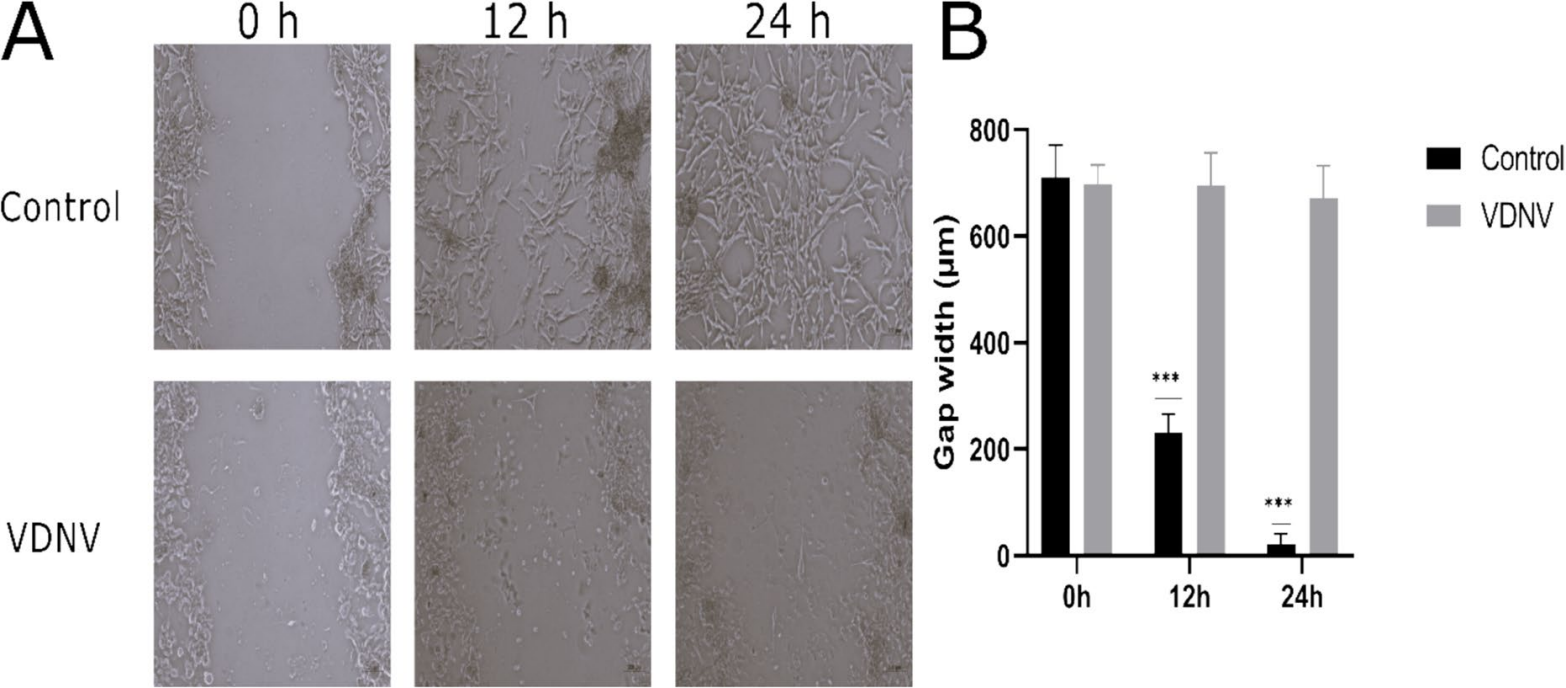
Wound healing analysis of U87MG cells. Images of wound closure (**A**) and corresponding quantitative analysis (**B**) were presented for both control and VDNV-treated groups at 0, 12, and 24 h. All experiments were conducted in three independent biological replicates (*n* = 3). ***p < 0.001

The effect of VDNVs on U87MG glioma cell migration was demonstrated qualitatively and quantitatively by wound healing assay. The impact of VDNVs added at IC50 value after physical wound creation was determined by measuring the distances between the scratches. Wound closure percentages at 0, 12, and 24 h were calculated. The scratch in the control group was completely closed by 24 h, while VDNV treatment inhibited cell migration over time (Fig. 3A). The closure rates were 96.33% for the control group and 3.09% for the VDNV group (Fig. 3B).

#### Determination of apoptosis

**Fig. 4:**
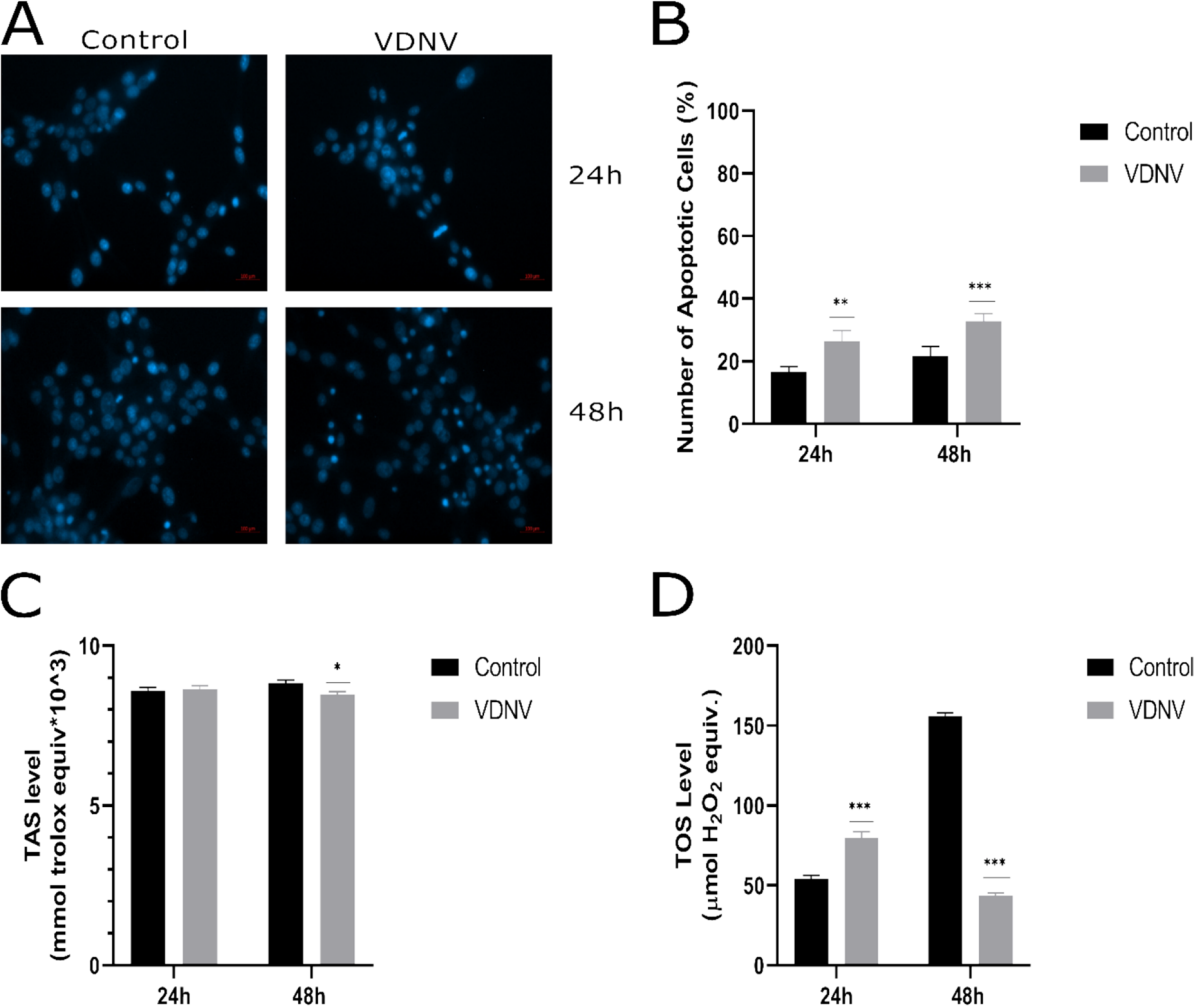
Apoptosis and antioxidant activity analyses of U87MG Cells. DAPI staining images of U87MG cells were captured (**A**) and quantitatively represented (**B**) at 24 and 48 h. TAS (**C**) and TOS (**D**) assays were conducted to evaluate the antioxidant activity of VDNVs. Studies included both control and VDNV-treated groups. All experiments were performed in three independent biological replicates (*n* = 3). *p < 0.05, **p < 0.01, ***p < 0.001

In the DAPI assay, less staining was observed in the control group compared to the VDNV-treated group. It was observed that nuclear staining in cells treated with the IC50 dose of VDNV at 24 and 48 h increased proportionally to the incubation time. This shows that VDNV treatment caused the U87MG cells to undergo apoptosis. While the apoptotic cell rate was 26% at 24 h, it was calculated as 17% in untreated control cells. In the 48 h, the rate was 21% in the control group and 33% in the VDNV group. In addition, when the 48-h results were compared with the 24-h results, an increase in the number of cells undergoing apoptosis was observed in both the control and VDNV groups (Fig. 4A-B).

#### TAS and TOS calculations

TAS value at the end of 24 h was 8.49 × 10^3^ mmol Trolox eqv. and 8.54 × 10^3^ mmol Trolox eqv. for the control and VDNV groups, respectively (*p* = 0.9118). At 48 h, it was 8.73 × 10^3^ mmol Trolox eqv. for the control group and 8.46 × 10^3^ mmol Trolox eqv. for the VDNV group (*p* = 0.0081). While the values ​​obtained at the end of 24 h were statistically insignificant, the data at 48 h were significant (Fig. 4C). TOS values showed statistical significance at both 24 and 48 h. The values for the control and VDNV groups ​​were 52.04-µmol H_2_0_2_ eqv. and 79.65-µmol H_2_0_2_ eqv. at the end of the 24 h, and 156.14-µmol H_2_O_2_ eqv. and 41.4-µmol H_2_O_2_ eqv. at the end of the 48 h, respectively (*p* < 0.001) (Fig. 4D).

## Discussion

This study is the first to demonstrate the presence of NVs smaller than 100 nm from *V. opulus* fruits through microscopic and quantitative analyses. *V. opulus* fruits were selected for NV isolation due to the previously elucidated molecular effects of its phenolic and organic compounds on brain-related diseases, as well as its significant anticancer and antioxidant properties. Resveratrol, a natural phytoalexin, exhibits neuroprotective properties and can penetrate the blood–brain barrier (BBB) at low levels [25]. This compound reduces cell survival in U87MG glioblastoma cells through phosphatase and tensin homolog and tumor protein p53-dependent mechanisms [26]. Naringenin, a flavonoid known for its anti-inflammatory properties and protection against oxidative damage, also traverses the BBB with ease. It demonstrates inhibitory effects on GB cells by downregulating matrix metalloproteinase (MMP)-2 and MMP-9 proteins, and attenuating p38 mitogen-activated protein kinase and extracellular signal-regulated kinase (ERK) activities [27]. Quercetin, another plant flavanol, is believed to enhance BBB permeability and reduce dysfunction in vivo by activating the canonical Wnt/β-catenin signaling pathway [28]. It inhibits migration and invasion in U87MG cells by suppressing glycogen synthase kinase-3β/β-catenin/zinc finger E-box-binding homeobox 1 signaling [29]. Catechins, antioxidant flavonoids, exhibit significant anticancer effects by inducing autophagy, arresting the cell cycle, and suppressing cell migration and invasion in glioma cells. These effects are attributed to the inhibition of the mitogen-activated protein kinase/ERK signaling pathway [30]. VDVNs are worth further investigation not only therapeutically due to the compounds contained in their source of origin but also as drug delivery systems. PDNVs contain remarkable features for this purpose. Primarily, PDNVs present several distinct advantages over synthetic nanoparticles. They are naturally occurring extracellular vesicle structures, extracted from mostly fruits, which confer biocompatibility and environmental sustainability [31] and are inherently enriched with bioactive molecules, including lipids, proteins, and nucleic acids that can potentiate their therapeutic and cargo applications. Conversely, synthetic nanoparticles are engineered through a myriad of chemical processes in laboratory settings, which occasionally incorporate toxic agents. Although synthetic nanoparticles can be precisely customized for specific purposes, they frequently encounter challenges related to biocompatibility and potential toxicity [32]. Moreover, the synthesis of synthetic nanoparticles generally necessitates considerable energy and resource expenditure, rendering them less sustainable compared to their plant-derived counterparts [31, 33].

One of the limitations of the study is that in vivo applications are lacking, and more comprehensive research is needed on the clinical use of VDNVs. A significant drawback is the unknown capability of VDNVs to cross the BBB. Overcoming this barrier is particularly challenging due to its highly specialized structure designed to protect the central nervous system. Due to its selective permeability feature, the BBB allows only certain substances to pass. While small molecules with a lipophilic structure can pass through the BBB, large molecules with a hydrophobic structure cannot [34]. The tight junctions between the endothelial cells that form the BBB close the gaps between the cells and prevent the passage of substances [35]. Although the BBB barrier is a difficult problem to overcome, some studies support exosomes as a promising approach. In a study conducted by Xu and colleagues, a nanoplatform containing exosomes derived from *Pueraria lobata* was synthesized and found to successfully cross the nasal mucosa and BBB and concentrate in the brain [36]. It is necessary to improve the physical properties of VDNVs and elucidate their molecular effects through more mechanistic studies. A comprehensive evaluation of the pharmacokinetics of VDNVs, especially their in vivo release kinetics, clearance, and determination of their safety profiles are also important. Such evaluations are vital for understanding the distribution, metabolism, and excretion of VDNVs in a biological system, thereby facilitating their integration into clinical practice. Despite the growing interest in PDNVs in clinical trials (clinicaltrials.gov, trials number: NCT04879810, NCT01668849, NCT01294072), their therapeutic efficacy in vivo remains insufficiently explored.

Obtaining such small NVs is difficult and requires optimization without UC which is considered the traditional way and gold standard in isolating PDNVs. Although the UC is very effective in NV isolation, it has many disadvantages. The method is not suitable for application in every laboratory due to the expensive equipment required, instability in the physical conditions (e.g., pH) of the solution during the process, very high speeds, aggregation formation, low yield, impurities, and structural deterioration [37]. Alternatively, polymer precipitation and SEC methods, following low-speed centrifugation, can be performed in any biology or chemistry laboratory and result in less damage to NV structures. There are some difficulties in the NV isolation method used in the study. The columns preferred were developed commercially for exosomes but can be used up to five times, with only 1 ml of sample applied at a time. This significantly reduced the amount of NVs obtained. In addition, the entire isolation process was completely manual, therefore, this restriction significantly impacts the concentration of NVs. Another difficulty encountered in the study is that NVs tend to form aggregations due to their nature. There are no physical/biochemical methods to protect PDNVs.

After isolating the NVs, as expected, aggregation formation was observed, reported as accumulated surface proteins [38, 39]. Small non-exosome artificial structures can also be formed in the NV isolation methods containing UC. In the optimized protocol in this study, the highest speed was 5000 × g, which caused minimal damage to NVs. Aggregations were detected with TEM and DLS. Although a centrifugal force of around 100,000 × g was not used in this study, the aggregation rate was below 10% and the presence of VDNVs was above 90%. While TEM analysis found NVs as small as 13 nm, DLS analysis showed the smallest NV size at 23.75 nm in this study. NVs isolated from grapefruit and tomato juices by differential UC showed diameters of 10–20 nm and 10–50 nm, respectively [40]. The average size of plant-based nanoparticles ranges from 180 to 200 nm. The zeta potential, which was − 2.87 mV, also shows the possibility of aggregation formation. It is known that particles with a zeta potential between − 10 mV and + 10 mV will rapidly agglomerate if their structures are not sterically protected [41]. According to literature research, the average zeta potential value for PDNVs is around -20 mV. The highest negative results were around − 30 mV [42] and the lowest value was − 0.6 mV [43]. Whether low or high-speed centrifugation affects the zeta potential of PDNVs remains unclear because various factors such as temperature, pH, ionic strength, and the nature of the NV source itself are also involved.

DLS analysis was performed to determine the size distribution of VDNVs. The analysis showed the smallest NV size at 23.75 nm, while TEM analysis found NVs as small as 13 nm in this study. NVs isolated from grapefruit and tomato juices by differential UC showed diameters of 10–20 nm and 10–50 nm, respectively [40]. The average size of plant-based nanoparticles is 180–200 nm. The use of the term “PDNV” for structures isolated with very high speeds or blade forces is a controversial issue. In these methods, small non-exosome artificial structures can also be formed. In the optimized protocol in this study, the highest speed was 5000 × g, which caused minimal damage to NVs.

In the study, VDNVs were used in cell culture studies by adjusting protein concentration. Proteins constitute a significant component of PDNVs and the concentration of these NVs exhibits a direct correlation with the protein content, implying that higher protein amounts correspond to increased NV quantities. Another investigation demonstrated the cellular effects of exosomes derived from lemon, fig, olive, and turnip by adjusting the protein concentration [44]. While methods like Nanoparticle Tracking Analysis (NTA) can determine PDNV concentration, calculating total protein concentration is easier and more accessible. NTA has significant disadvantages, including expensive equipment, time-consuming cleaning, and the potential for erroneous results at low concentrations [45]. The limitation of considering PDNV and total protein concentration as equivalent is that all exosomes in a solution are assumed to have the same protein content. Although both methods are popular, they cannot distinguish between subpopulations of extracellular vesicles. Therefore, it is important to obtain pure exosomes [46]. Reported PDNV concentration values vary widely, from 5.28 mg/ml (*Pueraria lobata* root derived) [47] to 104.87 μg/ml (goldenberry-derived) [32]. The most similar NV type to VDNVs in terms of protein concentration ratio is from *Carica papaya L.* fruit (1486–1761 μg/mL), but papaya PDNVs are two to three times larger (151.5–170.4 nm) [48] than VDNVs. Significant phenolic loss can occur from the juice to exosomes. The TPC of fresh *V. opulus* juice is 351.26-mg gallic acid eqv./100 mL [49]. This value is approximately 823 times higher than the TPC of VDNVs.

To evaluate the cellular effects of VDNVs, cytotoxicity, clonogenic cell survival, wound healing, apoptosis, TAS, and TOS experiments were conducted. In cytotoxicity analysis, IC50 calculation at 24 h was not feasible for HDF cells even at the highest dose (1000 μg/ml), because only 48% of the cells were inhibited. IC50 values indicated that VDNVs are 7.5 times more lethal to GB cells than to HDF cells after 48 h. Although some studies reported that PDNVs do not negatively affect cancer cells [50], most showed they are lethal to cancer cells. Doses and methods may vary, and the results can be influenced even when using the same fruit for isolation. Significant cytotoxic effects were seen for garlic-derived NVs up to 60 μg/mL in various cancer cell lines (U87, Panc-1a, Hep3B, SH-SY5Y, PC-3) in a study using a NV isolation method very similar to ours (Low-speed centrifugation + polymer-based precipitation buffer) [18]. Another study about the anti-glioma effects of ginseng-derived NVs UC, and sucrose density gradient methods were preferred. The IC50 value was 53 μg/mL for C6 glioma cells, and the cell proliferation remained at 100% level at 24 h. However, as seen in Fig. 2A, the proliferation rate below 50% in aggressively proliferating U87MG cells at both 24 and 48 h in this study.

HDF cells have a low response to VDNV treatment and require doses much higher than those applied to U87MG cells. In contrast, low IC50 values ​​of U87MG cells indicate an increased sensitivity to treatment, making them a more suitable model for evaluating the anticancer and antioxidant activity of VDNV. Consequently, to evaluate the efficacy of VDNV treatment and achieve more significant findings, subsequent experiments were conducted using only the U87MG cell line. In the clonogenic cell survival assay, VDNV-treated U87MG colonies exhibited a 2.7-fold reduction, whereas control group colonies increased as expected at 48 h (Fig. 2C-D). The observed reduction in colony formation within the VDNV-treated group highlights the inhibitory effect of VDNVs on U87MG cell proliferation. The number of colonies present at 48 h served as an indicator of cell survival rate, with a marked decrease in colony count in the VDNV-treated group due to successful inhibition and suppression of cell proliferation. Yang et al. isolated extracellular vesicles from lemon using a combined method containing an electrophoretic technique and dialysis bag and they observed significant decreases in cell proliferation in gastric cancer cell lines (AGS, BGC-823, SGC-7901) [51]. This demonstrates that plant-derived extracellular vesicles obtained without using UC can inhibit successfully cancer cells. Some studies showed the NVs isolated by UC cause less colony formation in cancer cells. Ginseng-derived NVs at concentrations of 60 μg/mL and 30 μg/mL were tested on lung cancer cell lines (A549, H1299) and showed lower colony formation compared to this study [52]. It is unclear whether the colony formation rate depends on the isolation method, the plant source, or both. Literature is insufficient for comprehensive comparisons of PDNVs, and studies are often shaped by IC50 values.

Wound healing experiments were performed with an artificially created scratch in a petri dish and the effect of VDNV treatment on gap closure was investigated. Due to the positive effect of FBS on cell proliferation and growth, these experiments were conducted under FBS-free conditions. FBS was intentionally excluded to prevent additional proliferation in high-growth U87MG cells. While the cells proliferated without FBS induction, the effect of NVs alone on wound healing was observed. In the control group, U87MG cells significantly closed the wound from 0 to 24 h (*p* < 0.05). On the contrary, in the VDNV group, no statistically significant difference was observed in the wound gap width at 12 and 24 h compared to 0 h. The wound did not close because VDNVs inhibited the proliferation and migration of U87MG cells. A study about *Momordica charantia*-derived NVs reduced migration in U251 GB cells at IC50 value (12 μg/mL) [53]. Different PDNVs inhibit cancer cells at various concentrations, suggesting that they have unique properties and mechanisms of action.

Cellular uptake of PDNVs is crucial for their therapeutic use, as each PDNV’s different lipid profile may target different cells. Phosphatidyl acid, a major PDNV lipid, is important for cellular uptake [54]. DAPI staining showed no significant difference between control and VDNV groups at 24 and 48 h, but apoptotic cells increased in VDNV-treated cancer cells. This slight increase in apoptotic cells at 48 h could be due to the low cellular uptake of VDNVs. Lipids and physical factors like temperature affect PDNV entry into cells [55].

TAS and TOS values were calculated to demonstrate the antioxidant properties of VDNVs. TAS analysis measures the total capacity of all antioxidants to neutralize free radicals and reactive oxygen species. In contrast, the TOS analysis quantifies the total amount of oxidants that contribute to pathological conditions. By simultaneously analyzing these two parameters, comprehensive information about the oxidative stress status of the cells was obtained. Cancer cells maintain a balance between reactive oxygen species and antioxidants to prevent damage. The decrease in TAS in VDNV-treated cancer cells indicates a reduction in antioxidant levels. In Fig. 4D, as expected, the TOS level increased in the control group, while it decreased in the VDNV-treated group. Major changes in TOS levels are not desired by cancer cells. In the VDNV group, it was observed that the TOS level decreased almost twofold from 24 to 48 h. It shows the strong antioxidant effect of VDNVs.

PDNVs show promising properties, but there is room for improvement. PDNVs exhibit several non-negligible disadvantages compared to synthetic nanoparticles. A significant limitation is the difficulty in standardizing their synthesis, which hinders the reproducibility and scalability of their properties for industrial applications [31]. Additionally, PDNVs often have low stability and short shelf life, complicating long-term storage and application. Also scaling up the isolation procedure and standardizing PDNV preparations presents several challenges. Traditional isolation methods such as UC often result in low yields, making large-scale production unfeasible. Furthermore, differences between sub-vesicle populations necessitate the establishment of standard protocols to ensure consistency among the PDNVs obtained. To consider VDNVs as delivery systems, in vivo circulation time, targeting specificity, tissue penetration ability, and effective release of the delivered active molecules should be taken into consideration carefully.

## Conclusion

In the study, NVs were derived from *V. opulus* fruits utilizing a cost-effective and readily accessible method. VDNVs have demonstrated significant cytotoxic effects on GB cells, indicating their potential in cancer treatment. Findings underscore their ability to inhibit cancer cell proliferation and migration. Continued research into the cellular effects of VDNVs is essential to maximize their therapeutic potential. PDNVs represent a relatively nascent field, with their physical, biochemical, and anticancer properties mainly unexplored. Their environmental friendliness and natural biocompatibility render them superior to synthetic nanoparticles. Advancements in PDNV isolation techniques hold great promise. By standardizing these processes, researchers can achieve more reliable and reproducible outcomes, fostering robust insights into PDNV biology and applications. Additionally, developing innovative filtering techniques tailored for PDNVs will be crucial in addressing the challenge of aggregate formation, without compromising sample concentration or requiring extensive isolations. Mechanistic studies will pave the way for understanding PDNV behavior, particularly in overcoming the BBB challenge in treating brain-related diseases such as GB. Further elucidation of the lipid profiles of PDNVs, which are potentially key to cellular internalization, could open new avenues for therapeutic interventions. This study is anticipated to significantly benefit researchers in the field, offering valuable insights and spurring new ideas in medical science.

## Data Availability

All generated data are included in this article.
